# Closing the delivery gaps in pediatric HIV care in Togo, West Africa: using the care delivery value chain framework to direct quality improvement

**DOI:** 10.1080/09540121.2016.1176678

**Published:** 2016-07-08

**Authors:** Kevin Fiori, Jennifer Schechter, Monica Dey, Sandra Braganza, Joseph Rhatigan, Spero Houndenou, Christophe Gbeleou, Emmanuel Palerbo, Elfamozo Tchangani, Andrew Lopez, Emily Bensen, Lisa R. Hirschhorn

**Affiliations:** ^a^ Hope Through Health, New York, NY, USA; ^b^ Department of Pediatrics, Children's Hospital at Montefiore, Albert Einstein College of Medicine, Bronx, NY, USA; ^c^ Department of Family and Social Medicine, Montefiore Medical Center, Albert Einstein College of Medicine, Bronx, NY, USA; ^d^ Hope Through Health/Association Espoir pour Demain, Kara, Togo; ^e^ Division of Global Health Equity, Brigham and Women’s Hospital, Boston, MA, USA; ^f^ Department of Global Health & Social Medicine, Harvard Medical School, Boston, MA, USA; ^g^ Ariadne Labs, Boston, MA, USA

**Keywords:** Pediatric HIV, care delivery value chain, global healthcare delivery, implementation science, Togo

## Abstract

Providing quality care for all children living with HIV/AIDS remains a global challenge and requires the development of new healthcare delivery strategies. The care delivery value chain (CDVC) is a framework that maps activities required to provide effective and responsive care for a patient with a particular disease across the continuum of care. By mapping activities along a value chain, the CDVC enables managers to better allocate resources, improve communication, and coordinate activities. We report on the successful application of the CDVC as a strategy to optimize care delivery and inform quality improvement (QI) efforts with the overall aim of improving care for Pediatric HIV patients in Togo, West Africa. Over the course of 12 months, 13 distinct QI activities in Pediatric HIV/AIDS care delivery were monitored, and 11 of those activities met or exceeded established targets. Examples included: increase in infants receiving routine polymerase chain reaction testing at 2 months (39–95%), increase in HIV exposed children receiving confirmatory HIV testing at 18 months (67–100%), and increase in patients receiving initial CD4 testing within 3 months of HIV diagnosis (67–100%). The CDVC was an effective approach for evaluating existing systems and prioritizing gaps in delivery for QI over the full cycle of Pediatric HIV/AIDS care in three specific ways: (1) facilitating the first comprehensive mapping of Pediatric HIV/AIDS services, (2) identifying gaps in available services, and (3) catalyzing the creation of a responsive QI plan. The CDVC provided a framework to drive meaningful, strategic action to improve Pediatric HIV care in Togo.

## Background

In 2000, less than 1% of people living with HIV in low- and middle-income countries had access to antiretroviral therapy (ART). Less than two decades later, approximately 40% of all HIV-infected patients were receiving treatment (UNAIDS, 2015). Yet in spite of this drastically improved access to ART and with it the potential to significantly decrease morbidity and mortality, only one in three of the 2.6 million children infected with HIV worldwide are currently receiving treatment (UNICEF, 2015). Inadequate access to effective HIV care, especially for pediatric populations, is far too common in resource-poor settings. This inequity in access, also known as the delivery gap, represents the inability to ensure provision of effective healthcare to the communities who are most in need. Such gaps are ubiquitous in settings such as northern Togo, where only 30% of eligible adults and children living with HIV are currently receiving ART (UNAIDS, 2014). Developing and implementing a strategy to close this delivery gap is one of the key challenges of ensuring care for all Pediatric patients living with HIV/AIDS.

An adaptation of the care delivery value chain (CDVC) may offer a solution to help address gaps in care delivery by facilitating better management of human resources and capital investments. Value chain analyses are a tool from management science used to map the complete sequence of activities required to deliver a product or service to consumers. Michael Porter and Elizabeth Teisberg applied the value chain analysis to the healthcare sector and developed the CDVC (Kim, Farmer, & Porter, 2013; Porter & Teisberg, 2006). The CDVC maps the discrete activities for a patient with a medical condition across the entire continuum of care. By mapping activities along a value chain, the CDVC allows program managers to better appreciate how resources are deployed and how information flows across activities in order to design and configure programs to maximize the value delivered to patients. The CDVC also enables the identification of gaps or opportunities to enhance value to patients by improving coordination and alignment of program activities within the local context. The CDVC provides managers with the knowledge they need to better allocate resources, improve communication flows, and coordinate activities across the continuum of care. Although mostly described for use in developed healthcare systems, the CDVC is equally relevant in resource-poor settings where vulnerable populations often experience fragmented healthcare services in the context of limited budgets to support service delivery (Kim et al., 2013; Rhatigan, Jain, Mukherjee, & Porter, 2009).

Hope Through Health (HTH) is a US-based not-for-profit organization that has been operating a community-based HIV treatment program in partnership with a local association and the Ministry of Health in northern Togo since 2004. In 2014 we applied the CDVC approach to map key activities needed to improve quality of services for children living with HIV/AIDS in northern Togo. Based on our experience implementing the CDVC framework to guide QI efforts, we argue that the CDVC framework has the potential to significantly advance the field of healthcare delivery science by offering a strategy to close healthcare delivery gaps, including gaps in pediatric HIV/AIDS treatment. In this case study, we describe our experience operationalizing the CDVC framework by integrating it into ongoing QI efforts with the overall aim of improving value for pediatric patients living with HIV/AIDS in northern Togo, West Africa.

## Methods

### Setting

As of December 2015, HTH and its partners provided ongoing comprehensive healthcare services to 1685 individuals living with HIV/AIDS in northern Togo, including 177 children enrolled in a specialized Pediatric HIV program. This public–private partnership is the largest Pediatric HIV program in Northern Togo.

### CDVC development

In order to inform the development of a CDVC for Pediatric HIV/AIDS in Northern Togo, we conducted a series of semi-structured facilitated discussions with key stakeholders, including HTH clinical and program staff, over a period of two weeks. These sessions served to map and assess existing care activities available to Pediatric HIV patients.

### Gap analysis

Using the CDVC, we analyzed available Pediatric HIV services, and then determined where gaps and/or deficiencies in care existed along the care continuum in the following discrete areas:

1. Prevention and Screening
2. Diagnosing and Staging
3. Pre-Antiretroviral Medical and Psychosocial Management
4. Intervening and ARV Initiation
5. Continuous Disease Management
6. Management of Complications and Clinical Deterioration

The team identified 22 unique gaps in care delivery within the aforementioned CDVC areas. These gaps were either activities that did not exist or were considered to be sub-par in quality and/or availability.

### Quality improvement plan

The team then designed and planned annual quality improvement programming based on identified gaps. A measurable indicator was identified for each gap, with a baseline measurement obtained, and an improvement target set. Each quality improvement objective was assigned a level of priority (first, second, or third). Fifteen of the 22 gaps were prioritized as quality improvement goals for a 12-month intervention period. The remaining seven gaps were determined not to be addressable within 12 months for reasons related to feasibility and finances. The majority of the quality improvement goals were concentrated in early stages of the care cycle including prevention, diagnosis, and treatment initiation (Table 1).

**Table 1.** Quality improvement objectives and associated target values, organized by CDVC area.

No.	CDVC Area	QI Objective and target value
1	Prevention and Screening	>90% of HIV+ pregnant women enrolled in pMTCT program initiate ART within first trimester
2	Prevention and Screening	<5% of HIV+ expecting mothers lost to follow-up
3	Prevention and Screening	>85% of infants screened for HIV by PCR method within first six weeks of life
4	Prevention and Screening	>80% of medicines on “essential medication” list available at all times
5	Diagnosing and Staging	>90% of mother/infant pairs identified as late for medical consultations receive monthly home visit
6	Diagnosing and Staging	>85% of HIV+ children screened for: (1) Tuberculosis and (2) Hepatitis B
7	Pre-antiretroviral Medical and Psychosocial Care	>95% infants (of mothers initially enrolled in pMTCT program) tested for HIV at 18 months
8	Pre-antiretroviral Medical and Psychosocial Care	Improved clinical integration developed between wrap-around support services and clinical program
9	Pre-antiretroviral Medical and Psychosocial Care	>75% of pediatric patients receive “disclosure counseling” by age 13
10	Intervening and ARV Initiation	>95% of patients receive initial CD4 test within 3 months of HIV diagnosis
11	Intervening and ARV Initiation	>95% of pediatric patients initiate ART within 3 months of date of eligibility for treatment
12	Intervening and ARV Initiation	>75% of “new caretakers” receive counseling sessions
13	Continuous Management	>85% of cohort of “high risk” patients adherent to ART
14	Management of Complications	>95% of “high risk” pediatric patients receive recommended periodicity for: (1) home visits and (2) medical consultations
15	Management of Complications	Quarterly morbidity and mortality sessions conducted

Note: PCR, polymerase chain reaction.

Over the next 12 months, the team directed local, dynamic strategies to identify root causes, develop, test, and iterate solutions using a Plan-Do-Study-Act (PDSA) cycle approach. Clinical staff submitted monthly reports to the HTH executive team with progress updates on bridging the prioritized gaps. Strategy development and iteration occurred through monthly discussions between clinical field-based and US-based technical staff. Data on each quality improvement goal were shared on a quarterly basis via a collaborative document shared among staff members.

## Results

### CDVC

**Figure 1.**
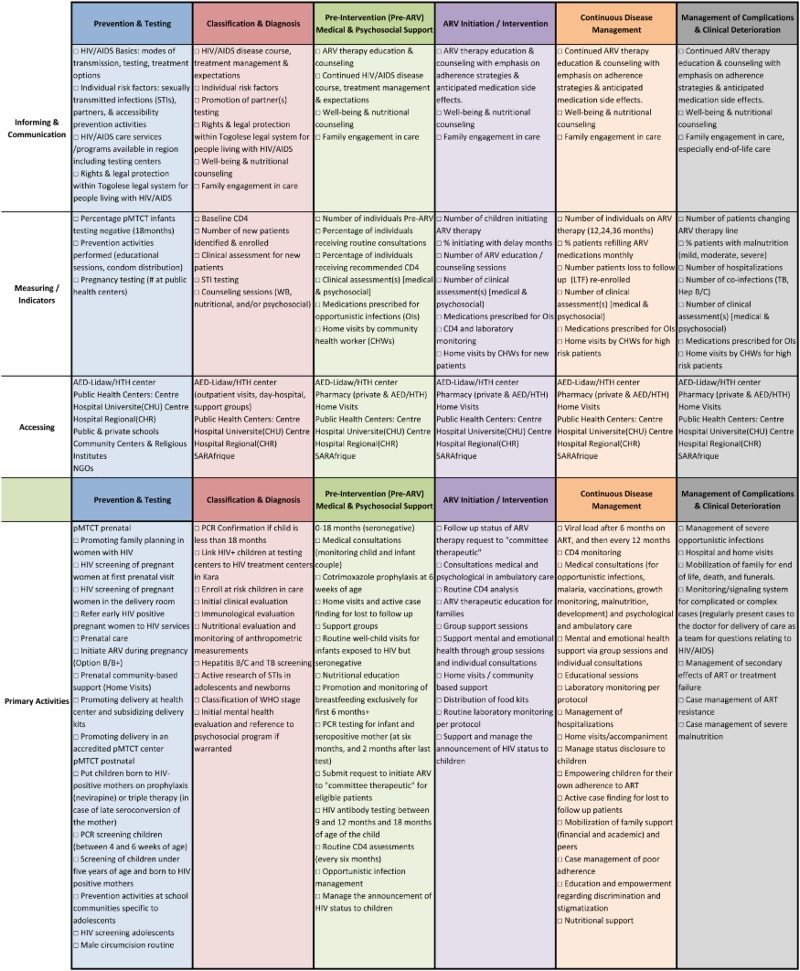
Care delivery value chain for pediatric HIV services in Kara, Togo in 2014–2015.

The first documented CDVC for Pediatric HIV/AIDS care in Kara, Togo was successfully drafted and used as a framework for mapping and improving service delivery (Figure 1) over a 12-month period. The framework was presented to partners including Ministry of Health collaborators.

### Quality improvement

Progress on each specific quality improvement goal was tracked over a 12-month period. Fifteen prioritized quality improvement objectives were initially monitored. During this 12-month period two goals were deemed unable to be tracked due to insufficiencies in required data collection systems. Of the remaining 13 unique objectives, 11 objectives achieved a priori targets and 2 target areas yielded minimal to no progress (Table 2).

**Table 2.** Accomplished QI objectives with target, baseline and year-end values.

No.	QI Prioritized objectives	Baseline	12 month
Target achieved			
1	>90% of HIV+ pregnant women enrolled in pMTCT program initiate ART within first trimester	20%	100%
2	<5% of HIV+ expecting mothers lost to follow-up	^a^	<1% (one pair loss-to-follow-up)
3	>85% of infants screened for HIV by PCR method within first six weeks of life	39%	95%
4	>80% of medicines on “essential medication” list available at all times	^a^	97%
5	>90% of mother/infant pairs identified as late for medical consultations receive monthly home visit	^a^	>98% (only one pair identified)
7	>95% infants (of mothers initially enrolled in pMTCT program) tested for HIV at 18 months	66.70%	100%
8	Improved clinical integration developed between wrap-around support services and clinical program	No plan	Plan created and implemented
10	>95% of patients receive initial CD4 test within 3 months of HIV diagnosis	66.70%	100%
12	>75% of “new caretakers” receive counseling sessions	<5%	95%
13	>85% of cohort of “high risk” patients adherent to ART	<5%	94%
14	>95% of “high risk” pediatric patients receive recommended periodicity for: (1) home visits and (2) medical consultations	(1) 85% (2) 97%	(1) 97% (2) 100%
Target not met			
11	>95% of pediatric patients initiate ART within 3 months of date of eligibility for treatment	60%	75%
15	Quarterly morbidity and mortality sessions conducted	2	1 over 12-month period
Insufficient data collection			
6	>85% of HIV+ children screened for: (1) Tuberculosis and (2) Hepatitis B	NA	NA
9	>75% of pediatric patients receive “disclosure counseling” by age 13	NA	NA

^Note: PCR, polymerase chain reaction.^

^a^Baseline data did not exist for this area, in process of QI efforts, developed strategy for capturing data.

## Discussion

**Figure 2.**
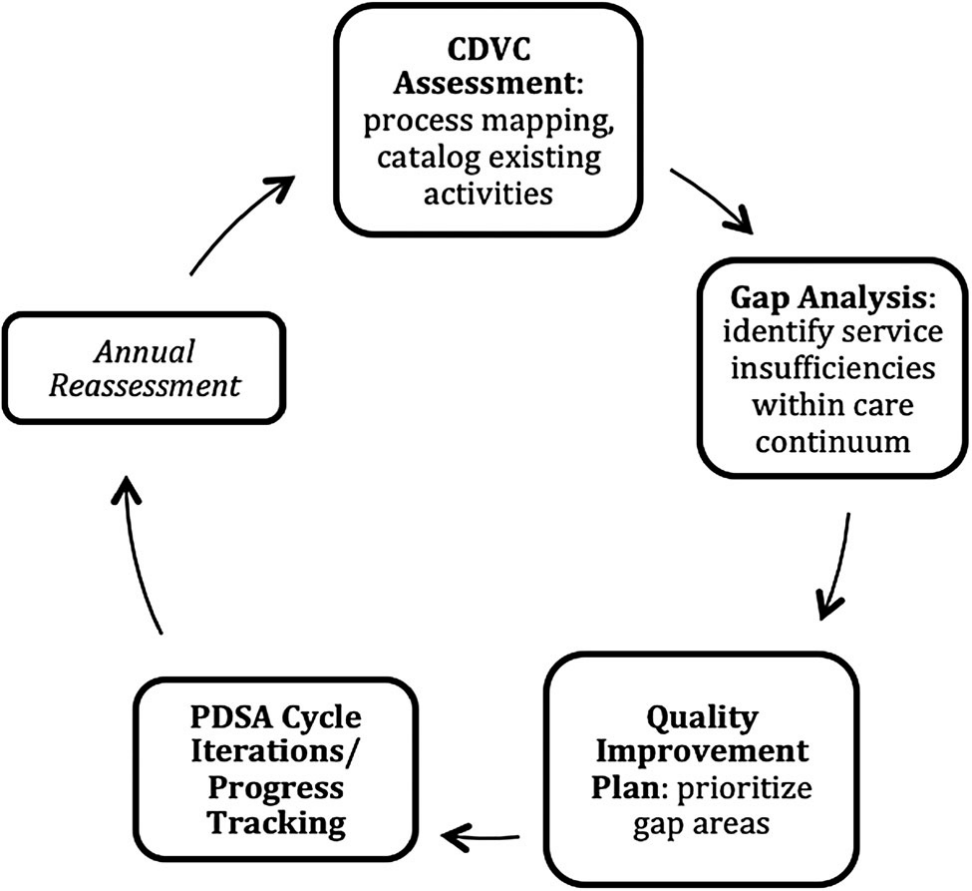
Schematic model for CDVC/quality improvement integration.

We found that utilizing a CDVC framework to identify and prioritize quality improvement activities was an effective strategy to improve care delivery over the full cycle of HIV/AIDS care for Pediatric patients in northern Togo. Using the CDVC framework resulted in the first comprehensive mapping of Pediatric HIV/AIDS services provided by a diverse set of actors, including the public sector, non-governmental organizations (NGOs), and community-based organizations (CBOs) specific to the Kara region of Togo. With respect to Pediatric HIV care, it enabled the clear definition of gaps or insufficiencies in currently available services across the full continuum of care. As a result, this approach catalyzed a strategic QI initiative based on identified care delivery gaps that resulted in concrete, measurable improvements in care delivery. Though significant progress was achieved for the majority of QI goals, the target areas that were not fully achieved will remain on the 2016 Pediatric HIV QI plan. This approach resulted in a new, strategic model for assessing care delivery activities, which then provided a guide for directing QI resources (Figure 2).

The first area of care for Pediatric HIV continuum involves the prevention of mother to child transmission (pMTCT), which experienced the most progress in our QI initiatives. The QI team, prioritizing the closure of delivery gaps for prevention, specifically targeted its efforts toward this domain of the CDVC, the primary prevention activity for pediatric HIV. The team developed and iterated systems of monitoring and evaluation for the pMTCT program and integrated communication between community health workers, midwife, medical assistants, and program coordinators. Due to their coordinated efforts, by the end of the year, ART was initiated for all women in the pMTCT program within their first trimester of pregnancy, closing the treatment gap for this specific population. In addition, through the development of a rigorous monitoring system, all mother–infant pairs were followed closely and actively until the infants reached 18 months of age and were declared seronegative for HIV. Similar coordinated efforts were conducted to achieve progress on the remaining CDVC/QI area objectives.

Though this approach was considered useful to our organization, it is not without limitations. The development of a CDVC in Northern Togo was prone to subjectivity from the participants as were the gaps identified. Additionally, the deficits in terms of data collection systems limited our ability to collect reliable data on some of the quality improvement initiatives implemented and to address all gaps identified. For example, the team initially identified 22 gaps, but was only able to address 60% of these gaps in the first 12 months. Lastly, we did not consider other process mapping approaches and therefore this case description cannot be considered a comparative study. Rather, this experience provides an example of how to adapt the CDVC framework to improve care delivery and management in a resource-poor setting in the hopes of having other organizations and communities learn from the experience. This approach may be particularly relevant to organizations with small or limited QI budgets.

In summary, the CDVC framework enabled the engagement of multiple voices, permitted the identification of previously unknown or invisible gaps and facilitated a strategic focus on multiple QI areas simultaneously, rather than sequentially, an advantage in resource-poor settings where many healthcare delivery challenges are interrelated. More work is required to optimize this approach, including systematizing the development of a CDVC for a medical condition, improving data collection systems, and strengthening local capacity to carry out PDSA QI iterations. Ultimately this process resulted in concrete, measurable actions that contributed to closing multiple delivery gaps over a 12-month period, thus catalyzing significant improvements in Pediatric HIV care for patients enrolled in HTH’s program in northern Togo. If adopted by others, this approach affords the opportunity to close delivery gaps in both inequity of access and quality of care for Pediatric patients living with HIV/AIDS.

## References

[CIT0001] Kim J. Y., Farmer P., Porter M. E. (2013). Redefining global health-care delivery. *The Lancet*.

[CIT0002] Porter M., Teisberg E. (2006). *Redefining health care: Crating value based competition on results*.

[CIT0003] Rhatigan J., Jain S., Mukherjee J., Porter M. (2009).

[CIT0004] UNAIDS (2014). http://www.unaids.org/sites/default/files/country/documents//TGO_narrative_report_2014.pdf.

[CIT0005] UNAIDS (2015). *AIDS by the numbers 2015*.

[CIT0006] UNICEF (2015). *Children & AIDS: 2015 statistical update.*.

